# Differential Pre-mRNA Splicing Regulates Nnat Isoforms in the Hypothalamus after Gastric Bypass Surgery in Mice

**DOI:** 10.1371/journal.pone.0059407

**Published:** 2013-03-20

**Authors:** William R. Scott, Cigdem Gelegen, Keval Chandarana, Efthimia Karra, Ahmed Yousseif, Chloé Amouyal, Agharul I. Choudhury, Fabrizio Andreelli, Dominic J. Withers, Rachel L. Batterham

**Affiliations:** 1 Centre for Obesity Research, Rayne Institute, Department of Medicine, University College London, London, United Kingdom; 2 Metabolic Signalling Group, Medical Research Council Clinical Sciences Centre, Imperial College London, London, United Kingdom; 3 Pitié-Salpêtrière Hospital, Assistance Publique-Hôpitaux de Paris, Cardiometabolic Department, Pierre et Marie Curie University, Institut National de la Santé et de la Recherche Médicale, Paris, France; St. Vincent’s Institute, Australia

## Abstract

**Background:**

Neuronatin (NNAT) is an endoplasmic reticulum proteolipid implicated in intracellular signalling. *Nnat* is highly-expressed in the hypothalamus, where it is acutely regulated by nutrients and leptin. *Nnat* pre-mRNA is differentially spliced to create *Nnat*-α and -β isoforms. Genetic variation of *NNAT* is associated with severe obesity. Currently, little is known about the long-term regulation of *Nnat*.

**Methods:**

Expression of *Nnat* isoforms were examined in the hypothalamus of mice in response to acute fast/feed, chronic caloric restriction, diet-induced obesity and modified gastric bypass surgery. *Nnat* expression was assessed in the central nervous system and gastrointestinal tissues. RTqPCR was used to determine isoform-specific expression of *Nnat* mRNA.

**Results:**

Hypothalamic expression of both *Nnat* isoforms was comparably decreased by overnight and 24-h fasting. *Nnat* expression was unaltered in diet-induced obesity, or subsequent switch to a calorie restricted diet. *Nnat* isoforms showed differential expression in the hypothalamus but not brainstem after bypass surgery. Hypothalamic *Nnat*-β expression was significantly reduced after bypass compared with sham surgery (P = 0.003), and was positively correlated with post-operative weight-loss (R^2^ = 0.38, P = 0.01). In contrast, *Nnat*-α expression was not suppressed after bypass surgery (P = 0.19), and expression did not correlate with reduction in weight after surgery (R^2^ = 0.06, P = 0.34). Hypothalamic expression of *Nnat*-β correlated weakly with circulating leptin, but neither isoform correlated with fasting gut hormone levels post- surgery. *Nnat* expression was detected in brainstem, brown-adipose tissue, stomach and small intestine.

**Conclusions:**

*Nnat* expression in hypothalamus is regulated by short-term nutrient availability, but unaltered by diet-induced obesity or calorie restriction. While Nnat isoforms in the hypothalamus are co-ordinately regulated by acute nutrient supply, after modified gastric bypass surgery *Nnat* isoforms show differential expression. These results raise the possibility that in the radically altered nutrient and hormonal milieu created by bypass surgery, resultant differential splicing of *Nnat* pre-mRNA may contribute to weight-loss.

## Introduction

Obesity is defined as excess body fat deposition and is a considerable global healthcare challenge. The World Health Organisation estimates that there are currently two billion obese or overweight adults worldwide [1]. Obesity is a leading risk factor for the development of type 2 diabetes, cardiovascular disease, cancer and dementia [2], [3], [4]. As the adoption of a Western energy-dense diet and sedentary lifestyle increases, particularly in low- and middle-income societies, the prevalence of obesity and burden of its complications are set to rise dramatically [5]. To compound this, available lifestyle and pharmaceutical therapies for obesity are ineffectual [6], [7], and bariatric surgery, the only effective and durable treatment, is reserved for the morbidly obese [8].

Excess body fat deposition occurs when energy intake from diet chronically outweighs energy expenditure [9]. Homeostatic centres in the hypothalamus and brainstem regulate appetite and energy expenditure according to short- and long-term signals of energy flux emanating from gastrointestinal, hepatic, pancreatic and adipose tissue, and from simultaneous higher order cortical cues [9], [10]. Within this multi-organ system a variety of genetic, molecular and physiological defects have been causally linked to obesity [11], [12], [13], [14], [15], however the precise biological mechanisms that favour long-term energy storage remain poorly characterised. Importantly, certain bariatric procedures like gastric bypass surgery lead to potent changes in appetite and energy expenditure with highly significant and durable weight-loss [16], [17], [18], most likely as a result of changes in gut hormones, such as ghrelin, peptide-YY (PYY) and glucagon-like peptide 1 (GLP1), as well as changes in neuronal and nutrient signals, that appear to override these intrinsic defects [8]. A greater understanding of the key regulators within this homeostatic circuitry and how they are modified after particular bariatric operations will be critical in the development of targeted strategies for the prevention and treatment of obesity.

Neuronatin (*NNAT*) is a paternally expressed imprinted gene residing within a large intron of the bladder cancer associated protein (*BLCAP*) gene [19], [20], variations in which are implicated in extremes of childhood and adult obesity [21]. The gene encodes two isoforms, NNAT-α of 81 and NNAT-β of 54 amino acids, derived by alternative splicing of the middle of three coding exons which eliminates a transmembrane domain [22]. Based upon shared sequence homology to proteolipids both isoforms are thought to regulate intracellular signalling via the endoplasmic reticulum (ER) calcium ATPase [23], [24]. In murine models, *Nnat* is primarily concerned with antenatal brain growth and development [22], [23], [25]. *Nnat*-α is expressed earlier than *Nnat*-β in gestational development implying isoform-specific regulation and function [22].

Recent studies also implicate *Nnat* in nutrient signalling and energy homeostasis. In mouse hypothalamus, *Nnat* expression is reduced following 48-h fast, specifically in the arcuate nucleus, dorsomedial hypothalamic nucleus (DMN), lateromedial hypothalamic nucleus and paraventricular nucleus (PVN) [21], [26]; is increased following peripheral administration of the satiety hormone leptin, though this is restricted to the DMN and PVN [21]; and co-localises in neurones containing important mediators of appetite control, including MCH, orexin and CART [21]. Thus hypothalamic *Nnat* expression is responsive to acute nutrient and leptin signalling in a neurone-specific fashion, and may mediate intracellular signalling to determine appetite.

In white adipose tissue (WAT), *Nnat* is over-expressed in obese and high-fat fed murine models, and is under-expressed in lipodystrophic and lean *S6kko* mice, as well as in mice fed conjugated linoleic acid to induce weight loss [27], [28], [29], [30]. In the pancreas, *Nnat* is expressed at high levels in islets cells [31], [32], where it is a target of the *NeuroD1* a transcription factor that is important for neuronal and endocrine cell differentiation and survival [30], [33]. *In vitro*, *Nnat* expression is associated with calcium-induced 3T3-L1 cell adipogenesis [28]; activation of PI3k, Erk, mTor and calcium signalling in medulloblastoma cells [34]; *Nfkb-*regulated inflammation in aortic endothelial cells [35]; and protection against mitochondrial toxins and ionophors in PC12 cells [36]. Similarly, in β-cell lines *Nnat* expression is associated with glucose-stimulated, calcium-induced insulin secretion [24], [33], whilst overexpression of the β-isoform may be concerned with ER stress and β-cell apoptosis [24]. In bioinformatic analyses, *Nnat* expression is highly correlated with expression of genes concerned with energy metabolism in the hypothalamus and WAT, in particular oxidative phosphorylation, and with inflammation in WAT alone [30].

Although previous studies may suggest a role for *Nnat* in acute appetite and energy homeostasis and in metabolic-inflammation the physiological function(s) of *Nnat* remain largely unknown. Further, there is limited data on the total or differential expression of *Nnat* isoforms in metabolic tissues in response to either acute or chronic changes in nutrient signalling. We therefore undertook studies to investigate the isoform-specific regulation of *Nnat* in the hypothalamus in response to acute nutrient fasting, high-fat diet-induced obesity, chronic dietary caloric restriction and modified gastric bypass surgery. These states were selected to represent a broad range of short- and long-term hormonal, nutrient and neuronal signals of energy flux, with the potential to regulate hypothalamic *Nnat* expression. We also examined expression of *Nnat* isoforms in other metabolic tissues involved in energy regulation.

## Results

### *Nnat* Expression in Central Nervous System (CNS), Adipose Tissue and Gastrointestinal (GI) Tract

**Figure 1:**
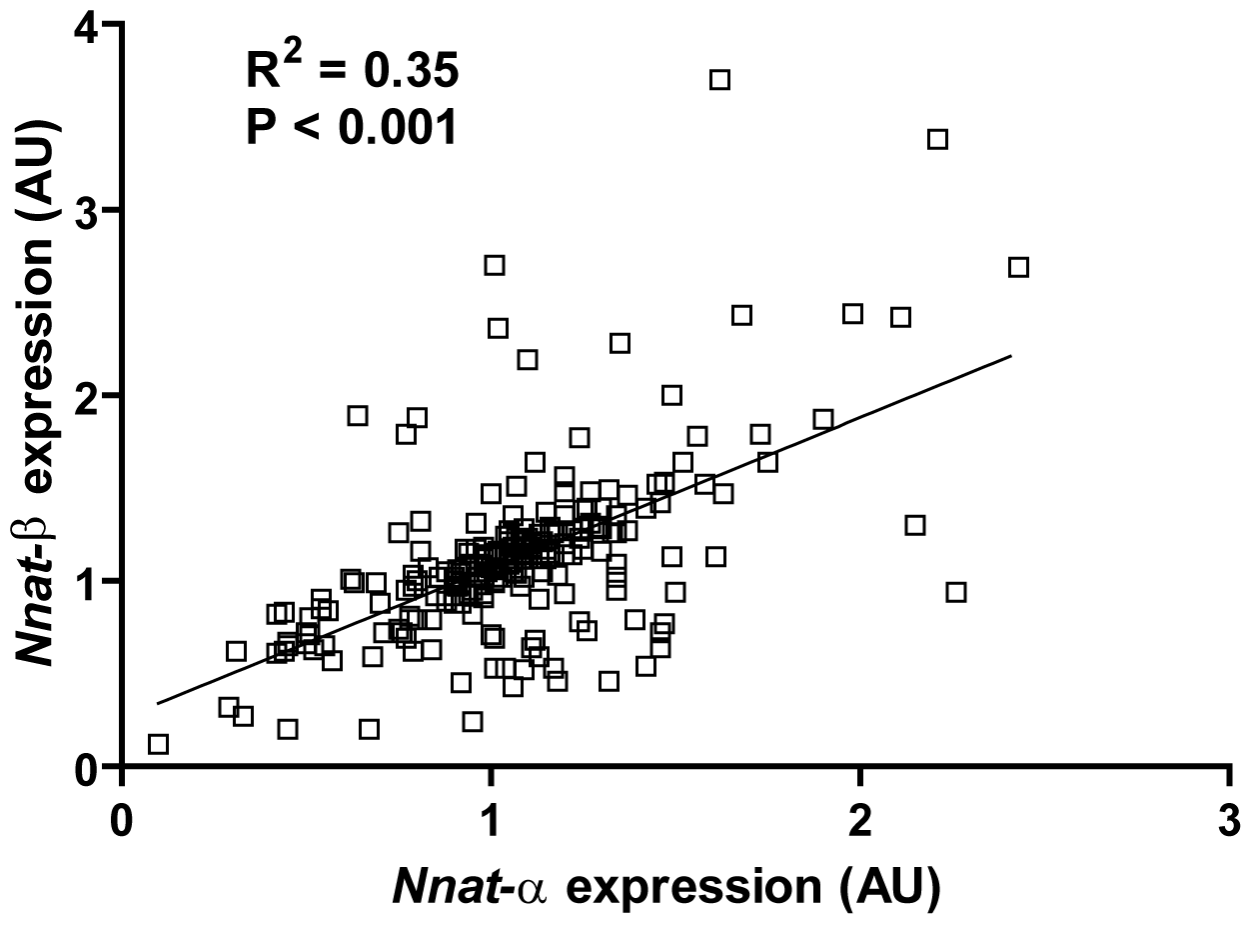
Relative expression between *Nnat* isoforms in metabolic tissues. *Nnat*-α expression was positively correlated with *Nnat*-β expression when compared in the hypothalamus and brainstem, white and brown adipose tissue, and stomach, duodenum, jejunum and ileum, implying shared and distinct regulatory factors (n = 229).

In adult mice both *Nnat*-α and -β isoforms were highly expressed in hypothalamus and WAT (Table 1). We also demonstrated abundant expression of both isoforms in brainstem, modest expression in brown adipose tissue (BAT), stomach and jejunum, and low expression in duodenum and ileum (Table 1). Relative *Nnat*-α and -β expression in all tissues under all conditions (including diet studies below) showed positive correlation (Figure 1; R^2^ = 0.35, P<0.001).

**Table 1:** Tissue *Nnat* expression.

	*Nnat*-α	*Nnat*-β
**Hypothalamus**	0.55	0.47
**Brainstem**	0.30	0.25
**WAT**	0.28	0.17
**BAT**	0.0050	0.0031
**Stomach**	0.0029	0.0023
**Duodenum**	0.00075	0.00016
**Jejunum**	0.0066	0.017
**Ileum**	0.00093	0.00099

RNA was extracted from tissue for n = 4 mice and pooled for measurement of *Nnat* expression; *Nnat* expression was standardised relative to ubiquitin (*Ubc*) expression (*Nnat*-α or -β/*Ubc*).

### Effect of Acute Nutritional Regulation on *Nnat* Expression

**Figure 2:**
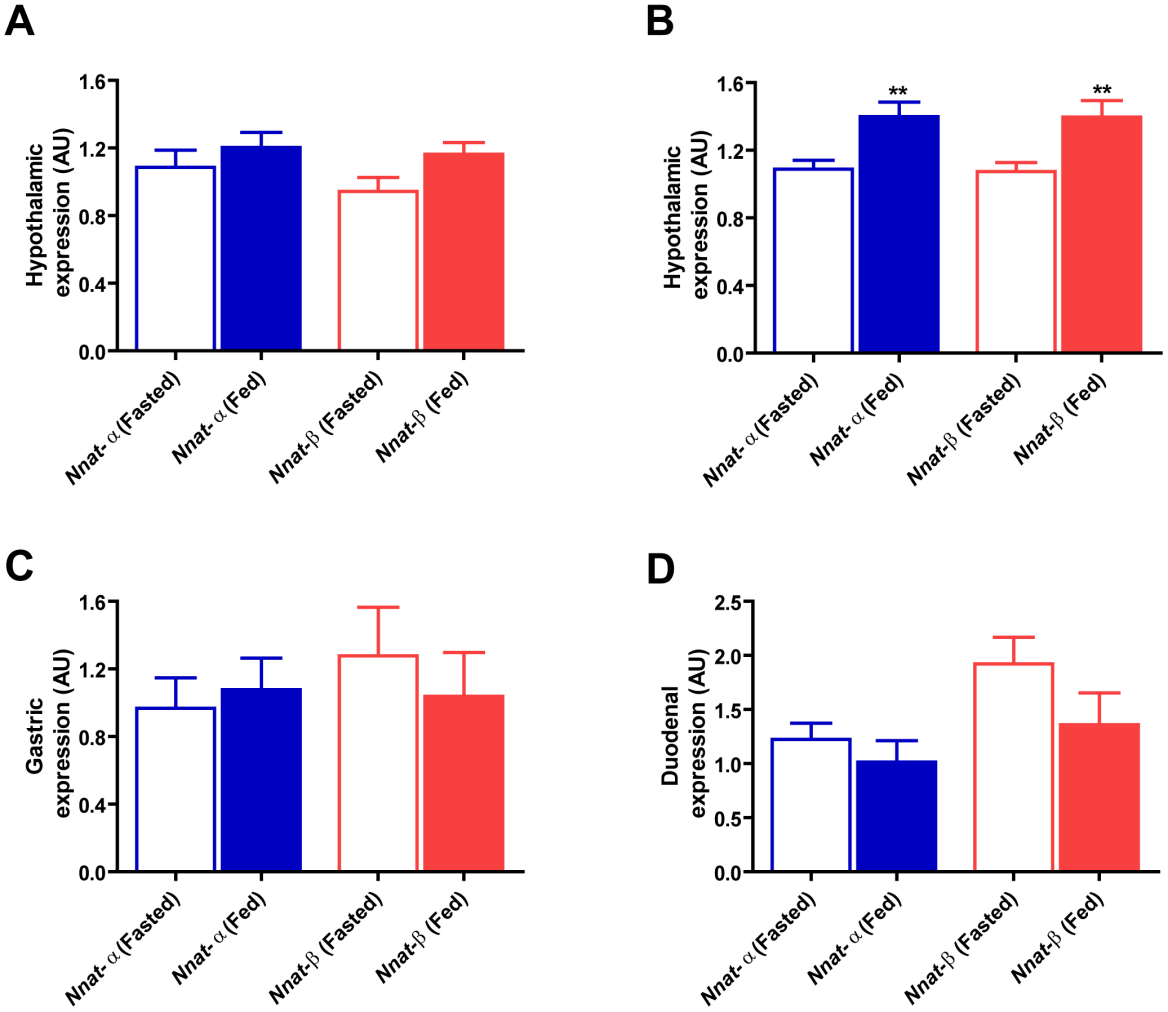
Expression of *Nnat* isoforms in response to fasting versus *ad-libitum* feeding. A) Hypothalamic *Nnat*-α and -β showed a non-significant reduction in response to overnight fasting (n = 10) when compared to *ad-libitum* fed counterparts (n = 10); B) but a significant reduction after 24-h fasting (n = 10) compared to feeding (n = 11), equivalent for both isoforms (**Nnat-α P = 0.005, **Nnat-β P = 0.007); C–D) *Nnat* isoforms did not show a consistent or significant change after overnight fasting in the stomach or duodenum (n = 10 fasted, n = 10 *ad-libitum* fed in both tissues); *key – AU = arbitrary units where Nnat expression was standardised using ubiquitin (Ubc) as a reference gene*.

We confirmed the influence of acute feeding state on global hypothalamic *Nnat* expression. Mean *Nnat*-α and -β expression levels were reduced in the fasted compared to *ad-libitum* fed state. This did not reach significance after overnight fast (Figure 2A; *t* test P = 0.397, P = 0.055; *Nnat*-α and -β respectively), but became highly significant after 24-h fast (Figure 2B; *t* test P = 0.005, P = 0.007; *Nnat*-α and -β respectively). In stomach and duodenum, mean expression did not differ significantly between overnight fast and *ad-libitum* fed groups, for either *Nnat*-α or -β (Figures 2C and 2D).

### Effect of Diet-induced Obesity (DIO) on Hypothalamic *Nnat* Expression

Adult mice fed high-fat diet for 4, 8, 12 and 16 weeks developed DIO. Final body-weight, increase in body-weight, and plasma leptin were significantly different in DIO groups compared to control groups maintained on standard dietary chow, except at week 4 for final body weight and week 8 for circulating leptin where the difference did not reach significance (Figure S1 A–C; Table S1). Body-weight increased between 4, 8, 12 and 16 weeks DIO, but less rapidly after 8 weeks (Figure S1A & S1B; Table S1; ANOVA overall significance levels P = 0.0001 for both final body-weight and delta body-weight). The difference in plasma leptin levels at different time points of DIO was overall significant (ANOVA overall significance level P = 0.038, though post-hoc comparisons did not show a significant difference between different time points). Mean global hypothalamic *Nnat* expression did not differ either between tiers of DIO or in comparison to controls, for either *Nnat*-α or -β (Figure S1D and S1E).

### Effect Of Caloric Restriction On Hypothalamic *Nnat* Expression

Mice with DIO and normal-weight counterparts were subjected to 4 weeks of caloric restriction. DIO mice were either maintained on high-fat diet, or switched to a diet of standard chow or caloric restriction. Normal-weight controls were exposed to equivalent dietary switches. This resulted in significant differences in final body-weight and change in body-weight between both DIO and control groups (Table 2A and 2B; DIO dietary switches, ANOVA overall significance P = 0.0001 for both final and delta body-weight; Control dietary switches, ANOVA overall significance P = 0.0001 for both final and delta body-weight). Fasting circulating plasma leptin was also significantly different in the DIO and control groups (Table 2A and B; DIO and Control dietary switches, ANOVA overall significance P = 0.0001). Mean hypothalamic *Nnat*-α and -β expression did not differ between DIO mice maintained on a high-fat diet, or switched to a diet of standard chow or calorie restricted (Figure S2A and S2B). Similarly, mean hypothalamic *Nnat* expression was comparable between control groups with parallel dietary switches (Figure S2A and S2B) and also did not differ when compared to the DIO groups, for either *Nnat*-α or -β (Figure S2A and S2B). *Nnat* isoforms showed no correlation with either weight reduction or fasting leptin after dietary caloric restriction (*Nnat*-α weight-loss R^2^ = 0.08, P = 0.08; leptin R^2^ = 0.05, P = 0.19; *Nnat-β* weight-loss R^2^ = 0.03, P = 0.29; leptin R^2^ = 0.05, P = 0.69).

**Table 2:** Change in body weight and leptin after chronic dietary switches.

	Control	Control-HF	Control-CR	HF-control	HF	HF-CR
**Body weight (g)**	28.8 (0.7)	33.6 (0.9)	20.5 (0.5)	30.5 (0.9)	35.1 (1.9)	26.2 (0.6)
**Delta body weight (g)**	−1.9 (0.4)	1.7 (0.4)	−8.0 (0.7)	−4.3 (0.7)	−1.3 (0.6)	−10.6 (1.1)
**Leptin (ng/ml)**	1.13 (0.16)	6.06 (0.16)	0.29 (0.05)	1.26 (0.25)	7.29 (1.91)	0.37 (0.08)
	**Final body-weight** **(P value)**		**Delta change in body-weight** **(P value)**		**Final plasma leptin** **(P value)**	
**Control ** ***vs*** **. Control-HF**	0.0001*		0.0001*		0.01*	
**Control ** ***vs*** **. Control-CR**	0.0001*		0.0001*		0.001*	
**Control-HF ** ***vs*** **. Control-CR**	0.0001*		0.0001*		0.01*	
**HF-control ** ***vs*** **. HF**	0.1		0.07		0.04*	
**HF-control ** ***vs*** **. HF-CR**	0.0001*		0.0001*		0.02*	
**HF ** ***vs*** **. HF-CR**	0.0001*		0.0001*		0.02*	

Control = standard dietary chow maintained, Control-HF = standard dietary chow switch to high-fat diet, Control-CR = standard dietary chow switch to caloric restriction; HF-Control = high-fat diet switch to standard dietary chow, HF = high-fat diet maintained, HF-CR = high-fat diet switch to caloric restriction; data presented as P value (post-hoc ANOVA).

### Differential Hypothalamic *Nnat* Expression After Bariatric Surgery, And Correlation Analyses

**Figure 3:**
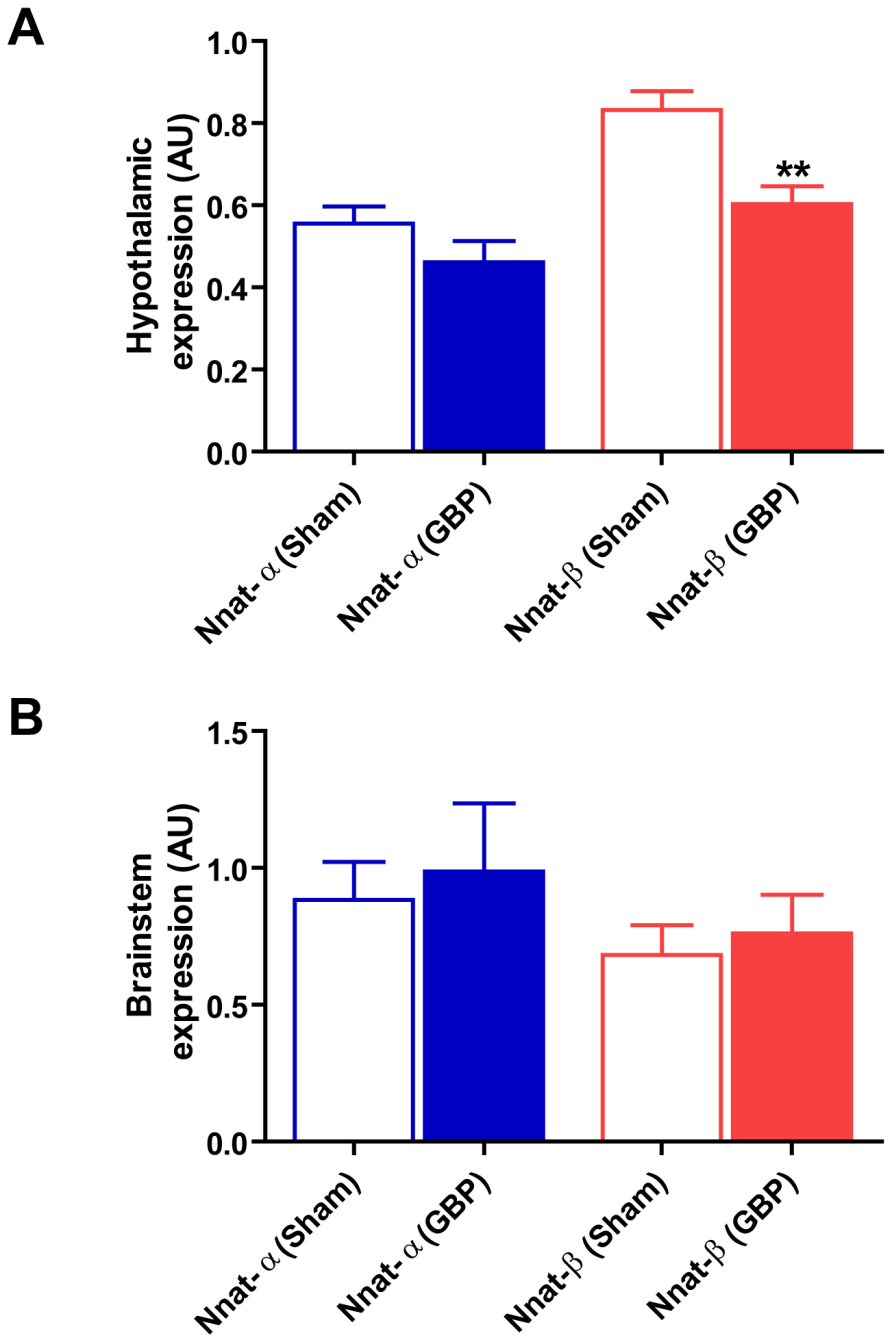
Hypothalamic and brainstem *Nnat* expression after modified gastric bypass *versus* sham surgery. A) *Nnat*-β showed a significant reduction in the hypothalamus (**P = 0.003) after modified gastric bypass (n = 8) compared to sham surgery (n = 7) whilst *Nnat*-α did not reduce significantly, consistent with a bypass-specific effect on *Nnat*-β expression; B) expression of *Nnat*-α and *Nnat*-β did not differ in the brainstem after modified gastric bypass (n = 8) compared to sham surgery (n = 7); *key – GBP = modified gastric bypass surgery, Sham = sham surgery, AU = arbitrary units where Nnat expression was standardised using an endogenous reference gene (ubiquitin (Ubc) for hypothalamus, hypoxanthine guanine phosphoriboribosyl transferase (Hprt) for brainstem).*

**Figure 4:**
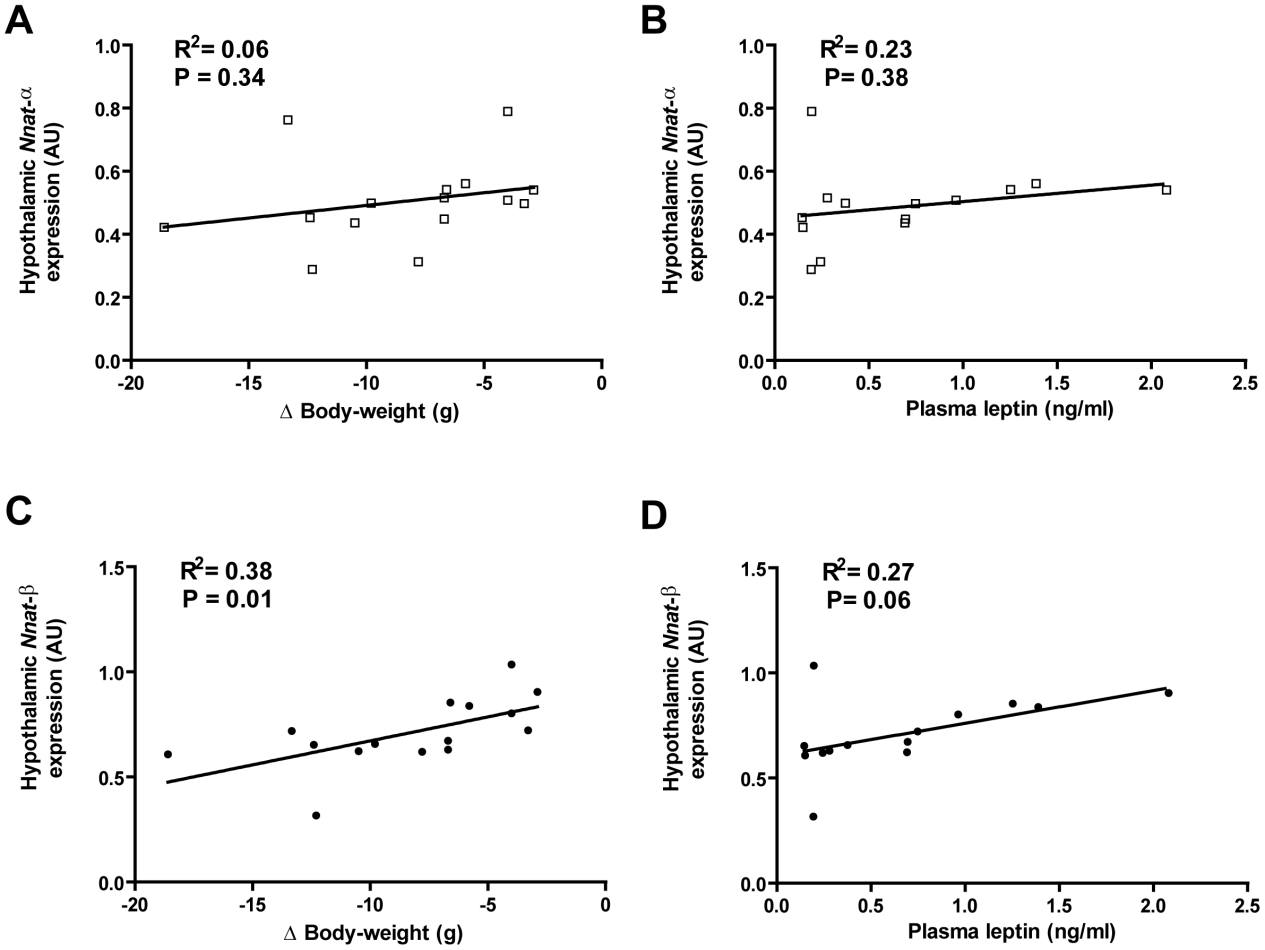
Isoform-specific *Nnat* expression in the hypothalamus after surgery in relation to weight-loss and circulating leptin. A–B) *Nnat*-α expression in the hypothalamus did not correlate with either change in body-weight or with circulating leptin after surgery; C–D) by contrast *Nnat*-β expression showed positive correlation with change in body-weight and weak positive correlation with circulating leptin after surgery; *key – Nnat-α expression shown with open squares, Nnat-β with filled circles, AU = arbitrary units where Nnat expression was standardised using ubiquitin (Ubc) as a reference gene*.

DIO mice subjected to modified gastric bypass surgery exhibited marked reduction in food intake throughout the post-operative period; food intake in sham mice reduced initially then returned to baseline pre-operative levels by post-operative Day 5 (Figure S3). Mice after bypass lost more weight and weighed significantly less at 10 days post-surgery compared to sham mice (Table 3). Fasting plasma leptin was significantly lower in the bypass versus sham group, whilst circulating fasting levels of the gut hormones acyl-ghrelin, total PYY and active-GLP1 were significantly higher (Table 3). *Nnat* isoforms showed differential expression in hypothalamus after bypass surgery (Figure 3A). Hypothalamic *Nnat*-β expression was significantly reduced after bypass compared with sham surgery (Figure 3A; *t* test P = 0.003). *Nnat*-α expression was not (Figure 3A; *t* test P = 0.188). *Nnat*-α expression did not correlate with reduction in weight after surgery (Figure 4A; R^2^ = 0.06, P = 0.34) or fasting plasma leptin (Figure 4B; R^2^ = 0.23, P = 0.38). However, *Nnat*-β showed positive correlation with weight reduction after surgery (Figure 4C; R^2^ = 0.38, P = 0.01) and weak correlation with fasting leptin concentration (Figure 4D; R^2^ = 0.27, P = 0.06). Neither *Nnat* isoform correlated with circulating fasting levels of the gut hormones acyl-ghrelin, total PYY and active-GLP1 after surgery (Table S2). *Nnat* expression was also analysed in the brainstem after bariatric and sham surgery. In brainstem *Nnat-*α and –β did not differ between bypass and sham counterparts (Figure 3B; *t* test P = 0.56, P = 0.51; *Nnat-*α and -β respectively).

**Table 3:** Changes in body-weight, leptin and gut hormones after surgery.

	Final body-weight (g)	Change in body-weight (g)	Plasma leptinlevel (ng/ml)	Plasma acyl-ghrelin level (pmol/l)	Plasma total PYY level (pmol/l)	Plasma active-GLP1 level (pmol/l)
**GBP surgery (n = 8)**	24.5 (1.2)	−11.4 (1.3)	0.27 (0.07)	1179 (258)	29.0 (5.1)	10.5 (2.9)
**Sham surgery (n = 7)**	31.1 (1.4)	−4.8 (0.6)	1.05 (0.23)	517 (83)	13.1 (1.8)	2.3 (0.4)
**GBS ** ***vs.*** ** Sham (P value)**	0.003*	0.0007*	0.004*	0.03*	0.01*	0.02*

GBP = modified gastric bypass surgery; data presented as Mean (Standard Error of Mean) or P value (*t* test).

## Discussion

Our key new finding is that *Nnat* isoforms are regulated by differential pre-mRNA splicing in the hypothalamus after modified gastric bypass surgery. We found that hypothalamic *Nnat*-β expression is significantly reduced after bypass compared with sham surgery, and is correlated with weight reduction and to a lesser extent with circulating fasting leptin after surgery. Alternatively, *Nnat*-α expression is unaltered after bypass compared to sham-operated mice and does not correlate with weight reduction or fasting plasma leptin. We found no relationship between fasting gut hormones and hypothalamic *Nnat* expression after surgery. In the brainstem expression of *Nnat*-α and -β is comparable after bypass and sham surgery. In the analogous experimental situation of dietary caloric restriction hypothalamic *Nnat* does not reduce either in total or differential expression, despite comparable total weight-loss and circulating leptin levels. Likewise, overall or differential expression of *Nnat* in the hypothalamus does not alter during the development of DIO. In contrast, Nnat-α and -β isoforms are comparably suppressed in the hypothalamus after an over-night and 24-h fast.

Differential expression of *Nnat* isoforms has been demonstrated previously, both during embryogenesis and in neuroendocrine cell lines. *Nnat*-α is first expressed in the brain of the mouse embryo at the time of neuroepithelial proliferation (embryonic days 7–10), whilst *Nnat*-β is expressed later during neurogenesis (embryonic days 11–14) [22]. In a murine pancreatic beta-cell line (MIN6) transfected with addition copies of the *Nnat* gene, the *Nnat-β* to *Nnat*-α ratio is increased proportionately to both glucose concentration and to length of glucose exposure [24]. The function of *Nnat* isoforms was tested in MIN6 cells by over-expression. At high glucose concentrations over-expression of *Nnat*-β induced ER stress and decreased the expression of genes important for beta-cell function, glucokinase (*Gck*), pancreas duodenum homeobox-1 (*Pdx-1*), and insulin [24]. Nnat-α did not have these effects [24]. *Pdx1* encodes a transcription factor necessary for pancreatic development and β-cell maturation. *Pdx1* is expressed in the developing neuroendocrine pancreas at a similar time to *Nnat* in the brain [37]. These reports indicate different roles for *Nnat*-α and *Nnat*-β in growth and differentiation during development, and in nutrient responsiveness within differentiated cells. Our observations, taken together with these previous findings, raise the possibility that factors within the milieu of signalling changes accompanying bypass may lead to suppression of the *Nnat*-β isoform through differential splicing of *Nnat* pre-mRNA. This in turn may alter neuroendocrine function in the hypothalamus in support of weight-loss.

Surgical reorganisation of gut anatomy causes changes in nutrient partitioning and absorption, with resultant adaptation of gut-derived hormonal, neuronal and nutrient signals. These signals are known to act in the hypothalamus, as well as in the brainstem and other CNS regions, and are considered in part to mediate the weight loss changes observed post-surgery [8]. We found no link between fasting gut hormone levels and differential hypothalamic expression of *Nnat* isoforms. This makes it unlikely that ghrelin, which acts to increase appetite primarily in the fasted state, plays a major role. Neither meal-stimulated gut hormone levels nor neuronal and nutrient signals were assessed; however all could mediate differential *Nnat* expression. Of note, post-prandial changes in PYY and GLP-1 show greater modification compared to fasted levels after bypass surgery [8]. Such changes could play a regulatory role if acting through a delayed mechanism, for example by direct or indirect modification of gene expression.

Alternatively, differential expression of *Nnat* isoforms in the hypothalamus may reflect changes in body-weight or food intake specific to bypass surgery. Total weight-loss *per-se* is unlikely to be responsible as we found similar total weight-loss after bypass and dietary caloric restriction, and *Nnat* expression was independent of weight-loss after dietary caloric restriction. However, mice at day 10 post-bypass are in an anorectic and accelerated weight-loss phase, whereas mice undergoing chronic dietary caloric restriction show more gradual weight reduction and exhibit increased hunger. These differences in energy status might mediate differential expression of *Nnat* isoforms after bypass; though this would appear to be hypothalamus-specific as brainstem expression is not comparably altered. Of note, circulating leptin is secreted proportionate to adipose tissue mass but also in relation to dynamic energy status irrespective of adipose tissue mass [38]. We observed equivalent circulating leptin levels after bypass surgery and dietary caloric restriction. Together these observations suggest that differences in energy balance do not fully account for differential effects on hypothalamic *Nnat* isoforms after bypass surgery.

Hypothalamic *Nnat* expression was assessed after overnight and 24-h fasts, compared to *ad-libitum* feeding; both *Nnat* isoforms showed a comparable modest reduction in expression after overnight fast and a larger reduction after 24-h fast. Published work has shown reduction in hypothalamic *Nnat* expression in response to prolonged 48-h fast [21],[26], and that leptin administration acutely increases *Nnat* in the DMH and PVN [21]. Previous studies find that *Nnat* expression is unaffected following 24-h fast in the cerebellum [26], a stress responsive tissue not concerned with energy balance, supporting the view that the changes in *Nnat* mRNA in the hypothalamus are a direct response to changes in nutrient or leptin signalling.

We investigated the effect of chronic high-fat feeding and DIO on hypothalamic *Nnat* expression and found no effect, despite significant differences in body-weight and circulating leptin between DIO and control mice. Thus, chronic nutrient excess and elevations in circulating leptin do not appreciably determine global hypothalamic *Nnat* expression, and hypothalamic *Nnat* expression is not perturbed in this model of obesity. Hypothalamic leptin resistance has been shown to develop as early as day 16 in DIO mice [39], and this may abrogate a leptin-dependent effect on hypothalamic *Nnat* expression in this model. Alternatively, we may miss hypothalamic nucleus-specific changes in *Nnat* expression since we examine only whole hypothalamus. These findings contrast with other metabolic tissues such as WAT and aortic endothelial cells where *Nnat* expression is increased in obesity and diabetes [30],[35].

The effect of chronic dietary caloric restriction on hypothalamic *Nnat* expression was also examined in mice with DIO and in normal-weight counterparts. We found no difference in *Nnat* expression in response to caloric restriction for either *Nnat* isoform, despite differences in final body-weight, change in body-weight and circulating leptin levels. In contrast to acute caloric restriction and modified gastric bypass surgery, chronic dietary caloric restriction leading to weight-loss with reductions in circulating leptin level has no appreciable effect on global hypothalamic *Nnat* expression. Importantly, these findings include mice with normal starting body-weight and intact hypothalamic leptin signalling, implying that hypothalamic leptin resistance does not account for unchanged *Nnat* expression either after chronic caloric restriction or in DIO.

To investigate the wider function of *Nnat* we examined its expression in a broad range of metabolic tissues. We identified *Nnat* expression at high levels in brainstem, substantial levels in BAT, stomach and jejunum, and lower levels in duodenum and ileum, and confirmed published results showing high levels of *Nnat* expression in WAT. When we compared relative expression of *Nnat* isoforms in these metabolic tissues we found modest positive correlation between *Nnat*-α and -β, suggesting potential differential isoforms expression in these tissues too. The role of overnight fast on *Nnat* expression in the stomach and duodenum was examined as these tissues have key acute nutrient sensing and signalling functions. No difference was found in stomach or duodenal *Nnat* expression after overnight fast when compared to *ad-libitum* fed counterparts; the role of 24-h fast was not examined in the absence of a directional effect equivalent to that found in the hypothalamus. While the overall functional significance of *Nnat* isoforms in metabolic tissues is likely to relate to total tissue expression levels, this may underestimate the local impact of *Nnat* within specialised neuroendocrine cells. This may be important in the GI tract, as in the pancreas, where neuroendocrine cells make up only a small proportion of total tissue and are the expected location of *Nnat* [31],[32]. The effect of acute fast on *Nnat* expression in the GI tract will need to be re-examined using localising techniques in enteroendocrine cells.

Our studies have several limitations. We focus on isoform-specific regulation of *Nnat* pre-RNA in global hypothalamus in response to states of acute and chronic energy flux, but do not investigate hypothalamic nucleus-specific changes in *Nnat*, or confirm our findings with protein expression. We also do not investigate in detail other brain regions involved in energy regulation. Our results demonstrate differential expression of hypothalamic *Nnat* isoforms after gastric bypass surgery. However, this finding is associative and the relative contribution of bypass-specific and other indirect factors remains to be established. We are able to make mechanistic inferences but do not delineate underlying biological mechanisms. Future studies are therefore required to localise changes in *Nnat* pre-RNA and protein isoforms in hypothalamic nuclei after bariatric surgery, DIO and dietary caloric restriction, as well as in other metabolic tissues, and to investigate precise causal processes within cellular systems and transgenic models.

### Summary And Conclusion

In the hypothalamus we demonstrate for the first time that differential pre-mRNA splicing preferentially suppresses *Nnat*-β in response to modified gastric bypass surgery, but not in response to the analogous situation of dietary caloric restriction. We further show that global *Nnat* expression is reduced in response short-term fast in the hypothalamus, though comparably for both isoforms. No effect on *Nnat* mRNA was discovered in response to chronic caloric excess and DIO. Our results indicate that the *Nnat* gene expression has shared (i.e. RNA transcription or turnover) and isoform-specific regulatory factors (i.e. differential pre-mRNA splicing). In addition our studies provide the first evidence that both *Nnat* isoforms are expressed in a broad range of metabolically active tissues, where the effect of nutritional status now needs to be examined further. Whilst the functional determinants of *Nnat* expression in the hypothalamus remain unclear, our findings raise the possibility that *Nnat* could mediate changes in appetite and energy expenditure during fasting/feeding and after bariatric surgery, via intracellular signalling changes. This position is strengthened by previously published work, which demonstrates co-localisation of *Nnat* in neuronal cells specifically expressing functional mediators of appetite control and energy expenditure [21]. Our study adds *Nnat* to the group of genes in which differential pre-mRNA splicing coordinates protein isoforms expression with potentially meaningful physiological consequences [40].

## Materials And Methods

### In Vivo Studies

All studies were performed in accordance with the Home Office Animal Procedures Act, UK (1986), project license PPL70/6648, and guidelines established by the European Convention for the Protection of Laboratory animals. All animals comprised male C57BL/6 mice (from Charles River U.K. Ltd), maintained in a pathogen-free environment at constant temperature with free water access, and subjected to 12-h light/dark cycle (0700–1900-h). Diets were obtained from Research Diets, New Brunswick, NJ USA (details specified below). Weight measurements were made using precision weighing balance (Sartorius, GE), accurate to 0.01 g.

For baseline expression studies in homeostatic tissues, adult mice without regulatory pre-conditions were used (n = 4, aged 8–16 weeks).

For acute nutritional studies, adult mice aged 8 weeks were randomised to *ad-libitum* standard dietary chow (composition 70% carbohydrate, 10% fat, 20% protein; Research Diets Catalogue D12450B), overnight fast (14-h duration) and 24-h fast groups (n = 10, n = 10, n = 11 respectively). All fasted mice were pre-conditioned, and experienced equivalent duration fast then re-feeding on three prior occasions.

For chronic high-fat diet and DIO studies, adult mice aged 8 weeks were randomised to *ad-libitum* high-fat diet (composition 35% carbohydrate, 45% fat, 20% protein; Research Diets Catalogue D12451) or standard dietary chow (composition above) groups, n = 40 in both. Body-weight was monitored weekly. After 4, 8, 12 and 16 weeks respectively ten mice from each dietary group were killed after 16-h fast. 16-h fasts were carried out in all chronic dietary studies (DIO; chronic dietary modulation with dietary switch; bariatric surgery) prior to termination. This minimises the confounding effect of acute feeding state, as systemic gut hormone levels may remain elevated up to 12-h after nutrient intake [41],[42].

For chronic dietary modulation with dietary switch studies, adult mice aged 8 weeks were randomised to *ad-libitum* high-fat diet (n = 30) and *ad-libitum* standard dietary chow (n = 30). After 16 weeks high-fat diet (DIO) mice were then randomised to one of three groups: i) maintained on this diet (n = 10); ii) switched to *ad-libitum* standard dietary chow (n = 10); or iii) switched to step-down caloric restriction (n = 10). Similarly control diet mice were randomised to one of three groups: i) maintained on standard dietary chow (n = 10); ii) switched to high-fat diet (n = 10); or iii) switched to step-down caloric restriction (n = 10). These diets were maintained for a further 4 weeks then mice were killed after 16-h fast. Body-weight was monitored weekly. Calorie restriction was carried out by step-down regime [43],[44]. Food intake and body-weight were assessed on a daily basis in DIO and control mice both maintained on *ad-libitum* standard dietary chow, to obtain measurements of food consumed per gram of body-weight. These measurements were then used to calculate food intake in DIO mice switched to caloric restriction and in control mice switched to caloric restriction respectively. For the step-down caloric restriction, an 80% restriction was applied in week 1, with 60% restriction in weeks 2–4. Food was given at onset of the dark phase (19.00-h) to maintain circadian rhythms.

For the bariatric surgery studies, adult mice aged 6 weeks were fed *ad-libitum* high-fat diet for 18 weeks, and then acclimatised for one week. After matching for body-weight, mice were assigned to modified gastric bypass or sham surgery (n = 8 for both groups). Procedures were carried out as previously described [45],[46]. Modified gastric bypass surgery comprised a midline laparotomy, ligature of the pyloric sphincter, and entero-gastric anastomosis between stomach fundus and mid-jejunum, thus excluding duodenum and proximal jejunum. Sham surgery comprised midline laparotomy, intestinal exposure and intestinal manipulation without transection, with duration corresponding to the bypass group. Daily post-operative monitoring for well-being was provided. One mouse in the sham group died post-operatively. On post-operative day 10 mice were re-weighed then killed, after a 16-h fast. This time point was selected for evaluation of factors contributing to weight-loss, rather than those resulting from marked changes in body-weight. Posthumously, the pyloric ligation was checked and was intact for all bypass subjects.

### Blood And Tissue Collection

Mice were killed using terminal anaesthesia. Blood was sampled by cardiac puncture using 29-gauge needle and syringe, after exposure of the chest cavity. For measurement of active GLP-1, dipeptidyl peptidase-IV inhibitor [10 µl/ml of blood] (Millipore, Watford, UK), was drawn into a chilled syringe before blood collection. For all other hormones blood was collected into an empty chilled syringe. Blood was transferred from syringes to tubes containing 0.5 M EDTA [50 µl/ml of blood], and aprotinin, [5000 Kallikrein inhibitor units (KIU)/ml of blood] (Trasylol; Bayer, UK). All samples were kept on ice until processing. Blood tubes were centrifuged for 15-mins at 10,000 rpm at 4°C. Plasma was aspirated by pipette and transferred to empty eppendorfs. For measurement of acyl-ghrelin, plasma was acidified by addition of 50 µl of 1 N hydrochloric acid per ml and 10 µl, 4-(2-Aminoethyl)-benzenesulfonyl fluoride hydrochloride (Fluka, Dorset, UK) 100 mg/ml was added to retard *ex-vivo* degradation. All samples were stored at −80°C until analysis.

All tissues were rapidly dissected and collected in cryovials (Nunc; Thermo Fisher, Roskilde, Denmark), then snap frozen in liquid nitrogen for storage at -80°C. Interscapular BAT and inguinal WAT were dissected. Stomach was divided from oesophagus and duodenum. The entire alimentary tract was removed from the body and fully extended. For duodenum, the first 3 cm of duodenum distal to the pyloric sphincter was dissected, a 3 cm section of jejunum (15 cm from the pyloric sphincter) was dissected, a 3 cm section of ileum proximal to the ileo-caecal valve. GI tissue was cleared of peritoneum and contents. After decapitation brain was removed. Hypothalamus and brainstem were dissected. The limits of the hypothalamus for dissection were: i) optic chiasma at the anterior border; ii) mammillary bodies at the posterior border; iii) the hypothalamic sulci at both lateral sides. Hypothalamic tissue was finally cut dorsally at 2 mm from the ventral face. Brainstem blocks were composed of pons and medulla regions of the hind brain and excluded cerebellum; dissection of the block started cranially at the level of intersection between midbrain and pons, and finished caudally at the level of intersection between pons and spinal cord.

### *Nnat* Mrna Expression Using Rtqpcr

Total RNA (tRNA) was extracted from homogenised whole tissue (including brainstem, hypothalamus, WAT, BAT, stomach, duodenum, jejunum and ileum) using a commercial extraction system (TRIzol reagent protocol). Quantity of RNA was measured using Nanodrop spectrometer, and integrity assessed by analysis of A260/A280 ratios. A two-tube technique was used for real-time quantitative PCR (RTqPCR). 2 µl of tRNA was reverse transcribed into cDNA under optimal conditions using Taqman reverse transcriptase reagents (Applied Biosystems, UK). RTqPCR was performed using proprietary sequence specific Taqman Gene Expression Assay FAM/TAMARA probes, specific for *Nnat*-α, *Nnat*-β, ubiquitin (*Ubc*) and hypoxanthine guanine phosphoriboribosyl transferase (*Hprt*), on an AbiPrism 7000HT instrument utilising automatically selected C_t_ values. *Ubc* was chosen as an endogenous reference gene to normalise expression data and account for differences in efficiency in all conditions except brainstem. In brainstem *Hprt* was used as the endogenous reference gene because of significant differences in *Ubc* expression between study groups. Target-specific standard curves were performed using 2-4-fold serial dilutions of template tRNA from tissue samples under investigation, and analysed to confirm efficiency, reproducibility and sensitivity, and to quantify the unknowns. Sample hypothalamic cDNA was diluted 1∶8, brainstem cDNA 1∶6, and stomach and duodenum cDNA 1∶5, to fit these standard curves. All samples were analysed in duplicate, with negative controls.

### Hormone Assays

Plasma leptin was measured as a marker of adiposity and chronic nutritional state. Plasma concentrations of leptin and active-GLP1 were measured using commercially available ELISA kits (Millipore, Watford, UK). Plasma acyl-ghrelin and total PYY were measured using commercially available radioimmunoassay (Millipore, Watford, UK). All samples were tested in duplicate.

### Statistics

For all expression studies, results were expressed as mean and standard error of the mean. Comparisons between *Nnat* expression, final body-weight, changes in body-weight and plasma leptin, were made using non-paired Student’s *t* test for comparing the means of two groups, and using one-way ANOVA for comparing the means of three or more groups. For one-way ANOVA post-hoc tests were performed in case a significant effect was detected (Bonferroni correction was used when the equality of variances assumption held, and Dunnett t3 correction was used otherwise). Significance was established at P<0.05. Linear regression analyses were performed to compare relationships between two continuous variables.

## Supporting Information

Figure S1 **Body-weight, circulating leptin and hypothalamic** ***Nnat*** **expression in response to diet-induced obesity.** A–C) Final body-weight, (delta) increase in body-weight, and plasma leptin were significantly different after 4, 8, 12, 16 weeks of high-fat feeding compared to control groups, except at week 4 for final body-weight and week 8 for plasma leptin where the difference did not reach significance; D-E) neither *Nnat*-α or -β expression was altered in the hypothalamus after 4, 8, 12, 16 weeks of high-fat feeding, either sequentially or compared to controls, *key – HF = high-fat diet, Control = standard dietary chow*.
(TIF)

Figure S2 **Hypothalamic** ***Nnat*** **expression in response to dietary caloric restriction.** A–B) *Nnat*-α and -β isoforms were equivalently expressed in DIO mice either maintained on high-fat diet or switched to standard dietary chow or caloric restriction, and in controls undergoing equivalent switches; *key – Control (standard dietary chow throughout, n = 10), Control-CR (standard dietary chow for 16 weeks then step-down caloric restriction for 4 weeks, n = 10), Control-HF (standard dietary chow for 16 weeks then switch to high-fat diet for 4 weeks, n = 10), HF (high-fat diet throughout, n = 10), HF-Control (high-fat diet for 16 week then switch to standard dietary chow for 4 weeks, n = 10) and HF-CR (high-fat diet for 16 weeks then step-down caloric restriction for 4 weeks, n = 10); AU = arbitrary units where Nnat expression was standardised using ubiquitin (Ubc) as a reference gene.*
(TIF)

Figure S3 **Food intake after modified gastric bypass versus sham surgery.** Mean 24-h caloric intake was significantly suppressed at Day 2 after surgery compared to pre-surgery baseline, in both bypass and sham groups; in the bypass group mean daily intake remained comparably suppressed up to day of termination (Day 10); by contrast, in the sham group mean daily intake returned to pre-surgery baseline by Day 5, and remained at this level until termination; food intake between the two groups was significantly different from Day 2; key – circles show mean food intake (g) per 24-h period in the modified gastric bypass group, squares show food mean intake (g) per 24-h period in the ad-libitum fed sham control group, standardised for mean body-weight (g); * ** *** represent P<0.05, <0.01, <0.001 respectively for within group comparisons; ### represents P<0.001 for between group comparisons.
(TIF)

Table S1 **Changes in body-weight and leptin in control and DIO mice.**
(DOCX)

Table S2 **Correlation of** ***Nnat*** **isoform expression and fasting gut hormone measures.**
(DOCX)
