# Supplementary figures and images for: Differential Pre-mRNA Splicing Regulates Nnat Isoforms in the Hypothalamus after Gastric Bypass Surgery in Mice
Source: PLoS One. 2013 Mar 20;8(3):e59407. doi: 10.1371/journal.pone.0059407 (PMC3603916; doi:10.1371/journal.pone.0059407)

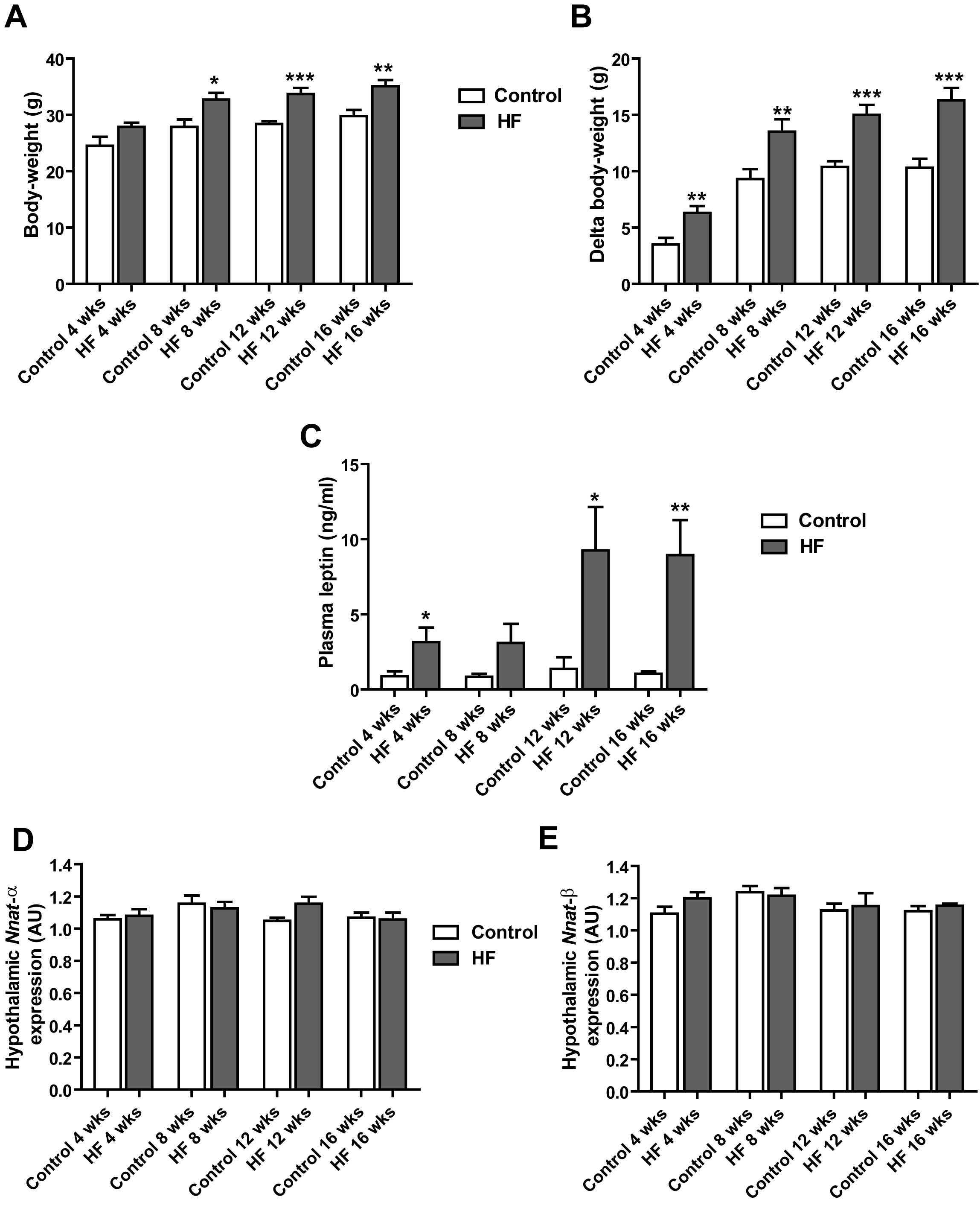

Supplement: Figure S1 — Body-weight, circulating leptin and hypothalamic Nnat expression in response to diet-induced obesity. A–C) Final body-weight, (delta) increase in body-weight, and plasma leptin were significantly different after 4, 8, 12, 16 weeks of high-fat feeding compared to control groups, except at week 4 for final body-weight and week 8 for plasma leptin where the difference did not reach significance; D-E) neither Nnat-α or -β expression was altered in the hypothalamus after 4, 8, 12, 16 weeks of high-fat feeding, either sequentially or compared to controls, key – HF = high-fat diet, Control = standard dietary chow. (TIF) [file pone.0059407.s001.tif]

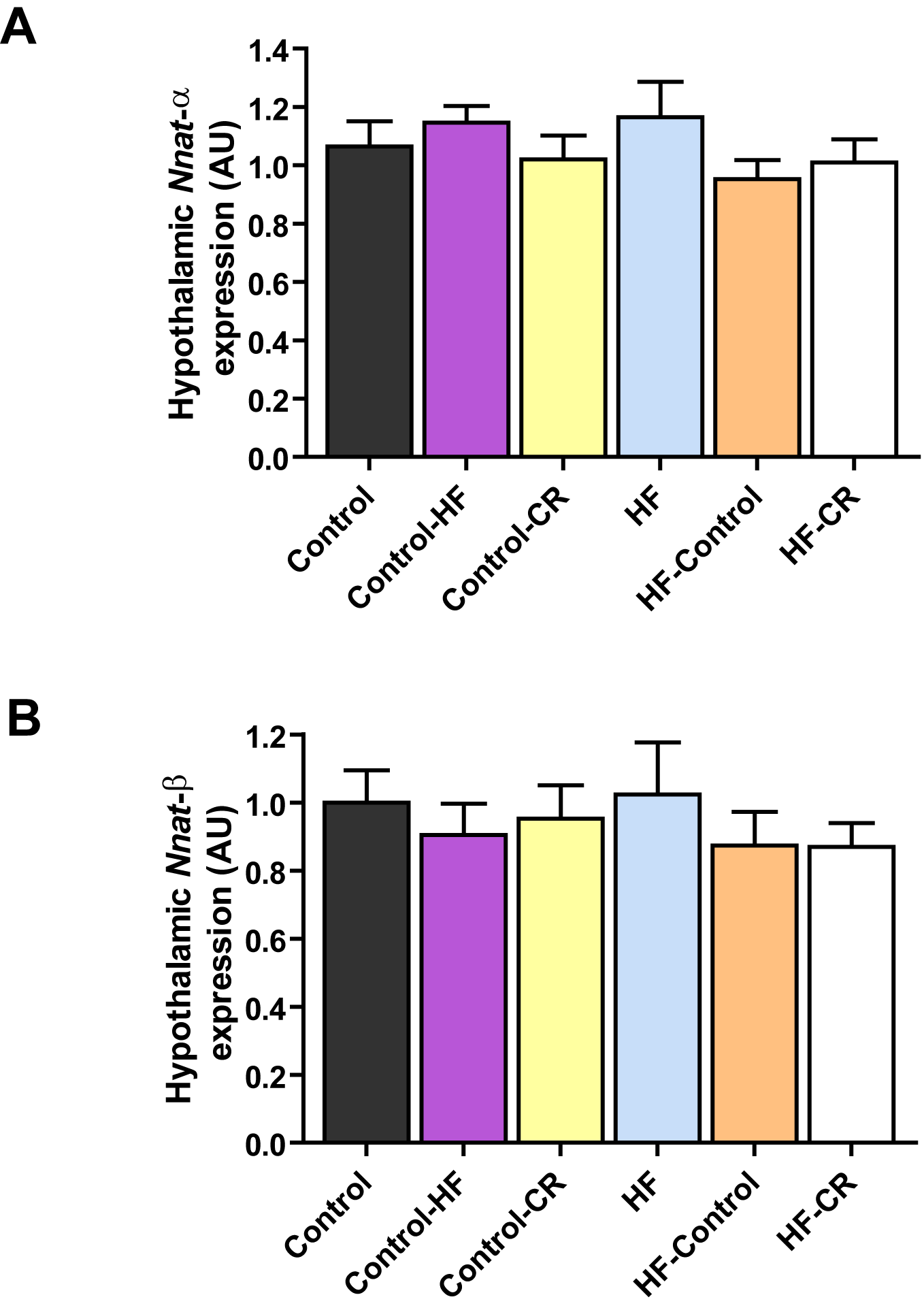

Supplement: Figure S2 — Hypothalamic Nnat expression in response to dietary caloric restriction. A–B) Nnat-α and -β isoforms were equivalently expressed in DIO mice either maintained on high-fat diet or switched to standard dietary chow or caloric restriction, and in controls undergoing equivalent switches; key – Control (standard dietary chow throughout, n = 10), Control-CR (standard dietary chow for 16 weeks then step-down caloric restriction for 4 weeks, n = 10), Control-HF (standard dietary chow for 16 weeks then switch to high-fat diet for 4 weeks, n = 10), HF (high-fat diet throughout, n = 10), HF-Control (high-fat diet for 16 week then switch to standard dietary chow for 4 weeks, n = 10) and HF-CR (high-fat diet for 16 weeks then step-down caloric restriction for 4 weeks, n = 10); AU = arbitrary units where Nnat expression was standardised using ubiquitin (Ubc) as a reference gene. (TIF) [file pone.0059407.s002.tif]

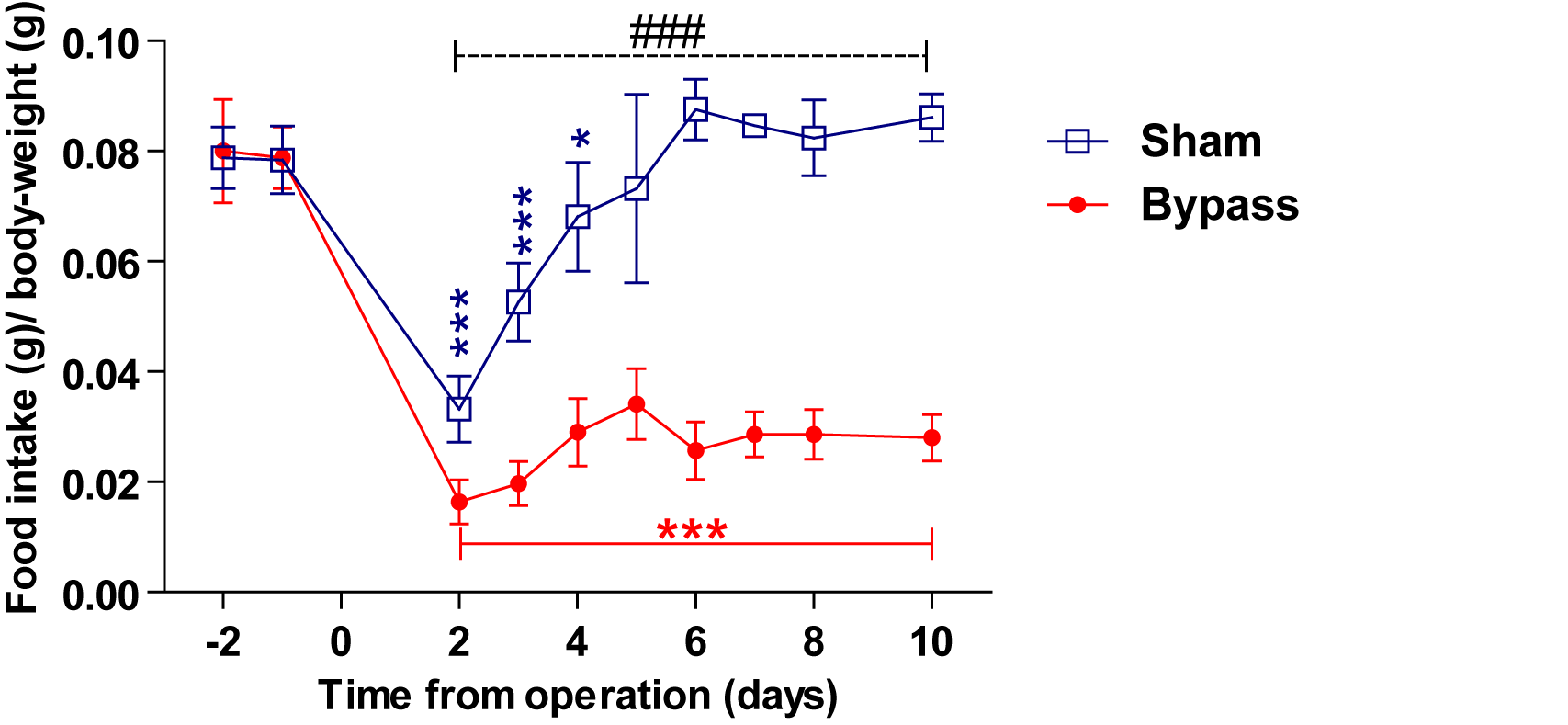

Supplement: Figure S3 — Food intake after modified gastric bypass versus sham surgery. Mean 24-h caloric intake was significantly suppressed at Day 2 after surgery compared to pre-surgery baseline, in both bypass and sham groups; in the bypass group mean daily intake remained comparably suppressed up to day of termination (Day 10); by contrast, in the sham group mean daily intake returned to pre-surgery baseline by Day 5, and remained at this level until termination; food intake between the two groups was significantly different from Day 2; key – circles show mean food intake (g) per 24-h period in the modified gastric bypass group, squares show food mean intake (g) per 24-h period in the ad-libitum fed sham control group, standardised for mean body-weight (g); * ** *** represent P<0.05, <0.01, <0.001 respectively for within group comparisons; ### represents P<0.001 for between group comparisons. (TIF) [file pone.0059407.s003.tif]
