# Supplementary material for: Differential Pre-mRNA Splicing Regulates Nnat Isoforms in the Hypothalamus after Gastric Bypass Surgery in Mice
Source: PLoS One. 2013 Mar 20;8(3):e59407. doi: 10.1371/journal.pone.0059407 (PMC3603916; doi:10.1371/journal.pone.0059407)
Supplement: Table S1 — Changes in body-weight and leptin in control and DIO mice. (DOCX) [file pone.0059407.s004.docx]

| **Table S1: Changes in body-weight and leptin in control and DIO mice.** | | | | | | | | | |
| --- | --- | --- | --- | --- | --- | --- | --- | --- | --- |
|  | **Body-weight (g)** | | | **Delta body-weight (g)** | | | **Leptin (ng/ml)** | | |
|  | **Control** | **HF** | **P value** | **Control** | **HF** | **P value** | **Control** | **HF** | **P value** |
| **4 wks (n=10)** | 24.5 (1.6) | 27.9 (0.7) | 0.07 | 3.5 (0.6) | 6.3 (0.6) | 0.003* | 0.88 (0.32) | 3.15 (0.97) | 0.04* |
| **8 wks (n=10)** | 27.9 (1.3) | 32.7 (1.2) | 0.01* | 9.3 (0.9) | 13.5 (1.1) | 0.01* | 0.84 (0.21) | 3.09 (1.28) | 0.1 |
| **12 wks (n=10)** | 28.4 (0.5) | 33.7 (1.1) | 0.001* | 10.4 (0.5) | 15.0 (0.9) | 0.001* | 1.37 (0.77) | 9.25 (2.89) | 0.04* |
| **16 wks (n=10)** | 29.8 (1.1) | 35.1 (1.1) | 0.004* | 10.3 (0.8) | 16.3 (1.1) | 0.0004* | 1.05 (0.16) | 8.95 (2.32) | 0.004* |
| **Control = Standard dietary chow, HF = High-fat diet resulting in diet-induced obesity; data presented as Mean (Standard Error of Mean) or P value (*t* test).** | | | | | | | | | |
