# Supplementary material for: Differential Pre-mRNA Splicing Regulates Nnat Isoforms in the Hypothalamus after Gastric Bypass Surgery in Mice
Source: PLoS One. 2013 Mar 20;8(3):e59407. doi: 10.1371/journal.pone.0059407 (PMC3603916; doi:10.1371/journal.pone.0059407)
Supplement: Table S2 — Correlation of Nnat isoform expression and fasting gut hormone measures. (DOCX) [file pone.0059407.s005.docx]

| **Table S2: Correlation of *Nnat* isoform expression and fasting gut hormone measures.** | | | | |
| --- | --- | --- | --- | --- |
|  | ***Nnat*-α correlation (R^2^)** | **P value** | ***Nnat*-β correlation (R^2^)** | **P value** |
| **Fasting acyl-ghrelin** | 0.06 | 0.36 | 0.05 | 0.41 |
| **Fasting total PYY** | 0.03 | 0.54 | 0.07 | 0.38 |
| **Fasting active-GLP1** | 0.09 | 0.73 | 0.47 | 0.63 |
| **PYY = Peptide-YY, GLP1 = Glucagon-like peptide 1; data presented as R^2^ and P value (linear regression).** | | | | |
|  |  |  |  |  |
